# The Guadalajara-Camptodactyly Syndrome-an unusual case

**DOI:** 10.1186/1546-0096-9-S1-P221

**Published:** 2011-09-14

**Authors:** RP Khubchandani, RP Hasija, M Dewoolkar

**Affiliations:** 1 Pediatric Rheumatology Clinic, Jaslok Hospital, Mumbai, India; 2 Pediatric Rheumatology Clinic, Jaslok Hospital, Mumbai, India; 3 Pediatric Rheumatology Clinic, Jaslok Hospital, Mumbai, India

## Background

Camptodactyly in seen in several syndromes, one such being the very rare Autosomal Recessive Guadalajara syndrome. Three distinct variants have been described (OMIM IDs 211910, 211920, % 611929).

## Aim

We describe our experience with a child with the Guadalajara syndrome who showed overlapping features of types I and II.

## Methods

A case description with a picture essay of the index case.

## Description

A 10 year old female, born of third degree consanguinity was referred for abnormal skeletal findings and suspected arthritis. On examination she had camptodactyly of all digits. Facial dysmorphism, camptodactyly, skeletal findings and subnormal intelligence were suggestive of the Guadalajara Syndrome. Facial dysmorphism (midfacial hypoplasia, hypertelorism, long neck, small, posteriorly rotated ears) and subnormal intelligence were compatible with Type I variant but muscle (gluteal hypoplasia), genital (labial hypoplasia) and skeletal findings (brachydactyly, simian creases, osteopenia and pelvic hypoplasia) were seen as has been described in the Type II variant. Other additional findings (not reported on OMIM to date), were a cervical rib, flattened thenar eminences, divarication of rectii and exomphalos. Short stature and microcephaly, known to be common to both the variants, were also noted.

## Conclusions

This child had overlapping features of both types of variants of the Guadalajara Syndrome. Her subnormal intelligence and craniofacial features were that of Type I while musculoskeletal findings were that of Type II. This leaves us to speculate that the two variants are probably not distinct entities but there exists a continuing spectrum in the syndrome.

